# Identification of Adolescent Menarche Status using Biplanar X-ray Images: A Deep Learning-based Method

**DOI:** 10.3390/bioengineering10070769

**Published:** 2023-06-26

**Authors:** Linzhen Xie, Tenghui Ge, Bin Xiao, Xiaoguang Han, Qi Zhang, Zhongning Xu, Da He, Wei Tian

**Affiliations:** 1 Department of Spine Surgery, Peking University Fourth School of Clinical Medicine, Beijing 100035, China; xlz2011@163.com (L.X.); gthgth315600@163.com (T.G.); xiaobin_spine@163.com (B.X.); hxg2119@163.com (X.H.); zhangqi7@pku.edu.cn (Q.Z.); xuzhongning111@pku.edu.cn (Z.X.); 2 Department of Spine Surgery, Beijing Jishuitan Hospital, Beijing 100035, China; 3 Research Unit of Intelligent Orthopedics, Chinese Academy of Medical Sciences, Beijing 100035, China

**Keywords:** menarche, adolescent, deep learning, X-rays, medical research

## Abstract

The purpose of this study is to develop an automated method for identifying the menarche status of adolescents based on EOS radiographs. We designed a deep-learning-based algorithm that contains a region of interest detection network and a classification network. The algorithm was trained and tested on a retrospective dataset of 738 adolescent EOS cases using a five-fold cross-validation strategy and was subsequently tested on a clinical validation set of 259 adolescent EOS cases. On the clinical validation set, our algorithm achieved accuracy of 0.942, macro precision of 0.933, macro recall of 0.938, and a macro F1-score of 0.935. The algorithm showed almost perfect performance in distinguishing between males and females, with the main classification errors found in females aged 12 to 14 years. Specifically for females, the algorithm had accuracy of 0.910, sensitivity of 0.943, and specificity of 0.855 in estimating menarche status, with an area under the curve of 0.959. The kappa value of the algorithm, in comparison to the actual situation, was 0.806, indicating strong agreement between the algorithm and the real-world scenario. This method can efficiently analyze EOS radiographs and identify the menarche status of adolescents. It is expected to become a routine clinical tool and provide references for doctors’ decisions under specific clinical conditions.

## 1. Introduction

Menarche in females marks a significant milestone in their sexual maturation, representing the initiation of ovulation and the emergence of reproductive capacity. The timing of menarche is subject to the influence of a diverse array of factors [1], including genetic predisposition, body mass index, genetic predisposition, climate variations, nutritional habits, and physical activity. Moreover, the occurrence of specific medical conditions, such as ovarian cysts [2], pituitary adenomas [3], thyroid dysfunction [4], and hepatitis [5], can also lead to changes in menarcheal age. Menarche is not only an important indicator for assessing female reproductive health and fertility but also plays an essential role in issues related to growth and development. The increase in sex hormone levels accompanying menarche plays a key role in the growth and maturation of bones [6]. Therefore, menarche is closely related to the development of the skeletal system, final height, and bone mineral content, and is one of the fundamental indicators for predicting the progress of developmental abnormalities [7,8]. In addition, extensive research has unveiled that the age of menarche also affects the long-term risk of developing breast cancer [9], endometrial cancer [10], cardiovascular disease [11], hypertension [12], and diabetes mellitus [13]. The menarcheal age serves as a potential prognostic factor for assessing the likelihood of developing these health conditions later in life.

Therefore, the status of menarche in adolescent females is a significant matter of interest for clinicians across various disciplines, including pediatrics, obstetrics, gynecology, orthopedics, endocrinology, and more. Currently, obtaining information on menarche primarily relies on questioning during clinical visits. However, it is noteworthy that adolescent females have the potential to withhold the disclosure of their menarche experiences from others due to feelings of fear, embarrassment, and confusion [14]. Furthermore, in actual clinical practice, menarche information may be missing as a result of various reasons, including medical negligence, patient forgetfulness, patient refusal to provide information, and loss of recording media. In the absence of menarche information, there is currently a lack of simple and practical alternative evaluation plans.

Menstruation is a fundamental component of the transition process from childhood to adolescence in girls. During this period, apart from the maturation of the reproductive function, there are certain patterns observed in the development of genital organ morphology, the emergence of secondary sexual characteristics, and the growth of the skeletal system. In theory, these development-related features have the potential to serve as indicators for inferring the menarche status, but currently, there are no available standards specifically for inferring menarche. For adolescents, full-spine/full-body EOS radiographs contain a wealth of radiological information related to growth and development. Nevertheless, the conventional method of statistically analyzing and synthesizing the image data from these X-ray images is cumbersome and impractical. To our knowledge, there have been no previous reports that utilize radiological information from X-ray imaging to infer menarche status.

A rise in artificial intelligence in some medical image recognition tasks has led to distinguishing between benign and malignant skin lesions based on surface photographs, performing diagnosis and severity classification of cataracts quantitatively using slit lamp and retroillumination photographs, and diagnosing chronic otitis media based on CT images. Ingeniously designed methods developed with deep learning techniques have approached or even surpassed the level of clinical doctors [15,16,17,18]. From a theoretical perspective, deep learning approaches, represented by convolutional neural networks, have demonstrated remarkable capabilities in extracting and combining graphical features. This ability enables them to extract meaningful information from complex visual inputs at different levels and perform comprehensive inference to accomplish corresponding downstream visual tasks [19]. Well-designed deep learning methods are expected to address the problem of the inability to objectively evaluate menarche status and provide practical assistance to doctors in clinical practice. Therefore, the purpose of this study is to develop a deep-learning-based method that can automatically identify the menarche status of adolescent females based on EOS X-ray images. To expand the scope of application, we added the classification of “males” in the design to make this method applicable to all adolescent populations.

## 2. Materials and Methods

**Figure 1:**
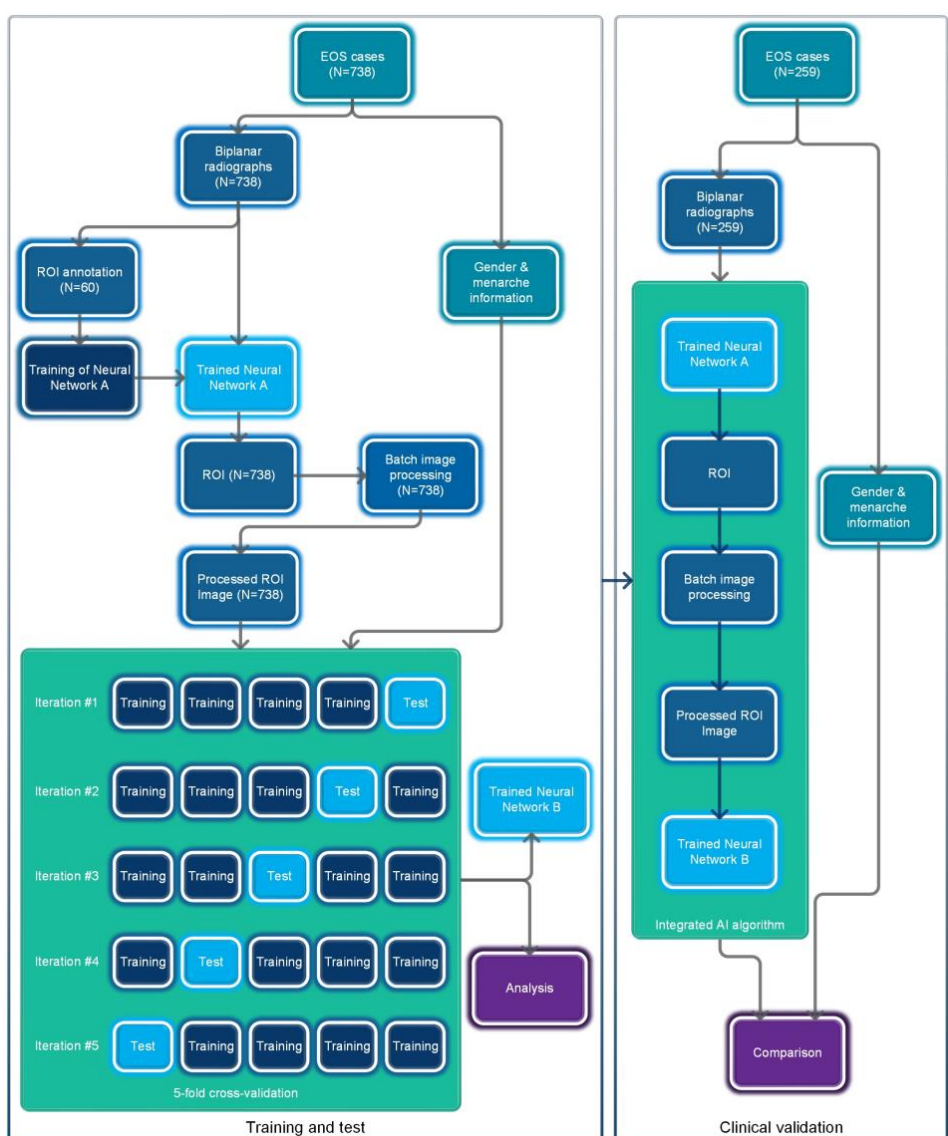
Flowchart of the overall process for this study.

The overall flowchart of this study is shown in Figure 1. This study utilized a retrospective data collection method and did not apply any artificial intervention measures to the subjects during the research process. The Ethics Committee of Beijing Jishuitan Hospital approved this study and waived the requirement for informed consent.

### 2.1. Data Collection

We retrospectively screened EOS cases using our institution’s picture archiving and communication system combined with the electronic medical record system. The specific inclusion criteria were as follows: (1) Age between 9 and 18 years at the time of examination. (2) East Asian. (3) Full-spine or full-body EOS radiography. (4) Complete and unobstructed image. (5) Complete frontal and lateral views. (6) For females, records regarding menarche were available. (7) No severe skeletal or muscular system deformities. (8) No intersex conditions. (9) No duplicates.

**Figure 2:**
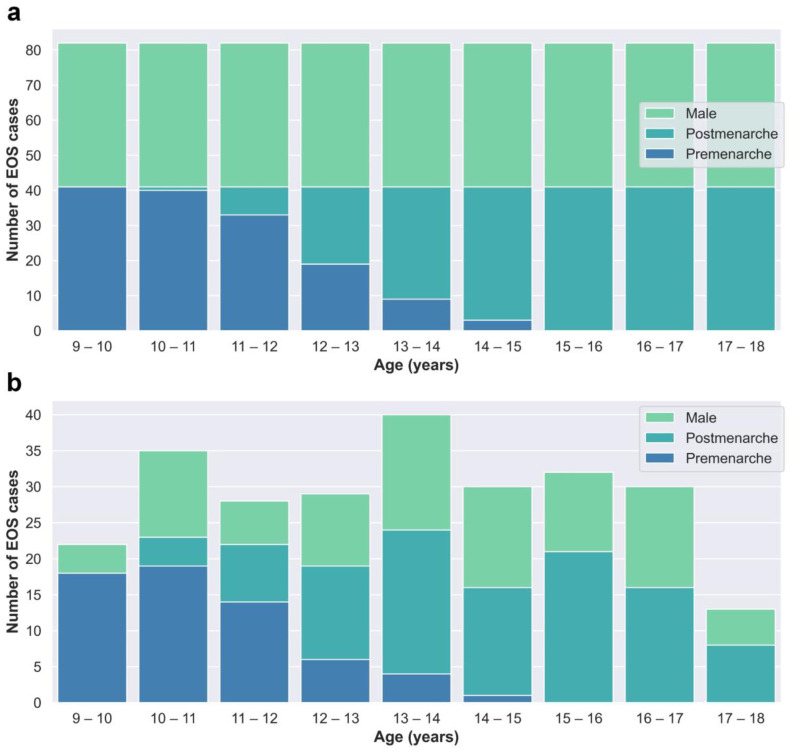
(**a**) Age–category distribution of Dataset A. (**b**) Age–category distribution of Dataset B.

The cases in the training and test dataset (Dataset A) were derived from patients who underwent EOS examinations at Beijing Jishuitan Hospital from January 2020 to March 2022. Segregated groups were established for males and females based on age, with a total of 18 groups designated for each year of age. Collection for each group stopped once it reached 41 cases, resulting in a total of 738 cases collected (Figure 2a).

The cases in the clinical validation dataset (Dataset B) were consecutively retrospectively collected from outpatient cases who underwent EOS examinations at Beijing Jishuitan Hospital from May 2022 to December 2022 to reflect real clinical situations. Dataset B included a total of 259 EOS cases (Figure 2b).

### 2.2. Neural Network A: Detection Network for ROI

**Figure 3:**
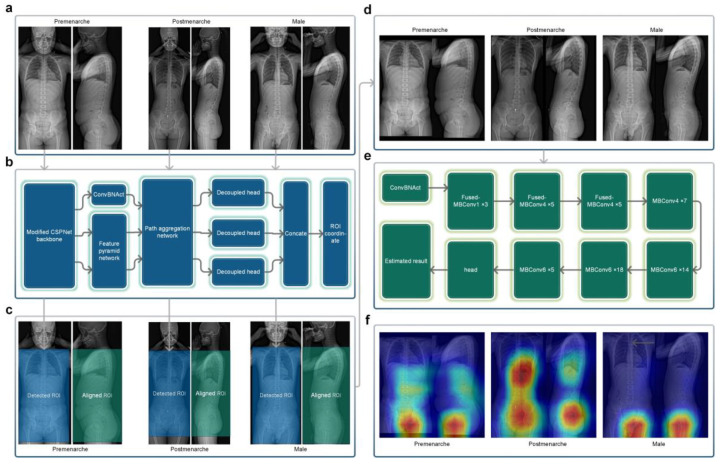
(**a**) Original EOS images of the three categories. (**b**) The structure of Neural Network A. (**c**) Detected frontal regions of interest and corresponding lateral aligned regions. (**d**) Stitched regions of interest images. (**e**) The structure of Neural Network B. (**f**) Class activation maps of the three categories. The regions in closer proximity to the red color represent a progressively more significant role played by those regions in the process of reasoning.

In order to maximize the use of effective radiological information in X-ray images, we selected a rectangular region that included the trunk from the level of the first thoracic vertebrae to the lower trochanter level as the region of interest (ROI) for the frontal radiograph X-ray images. Sixty EOS frontal radiographs were randomly selected from Dataset A for manual annotation of the ROI, which was performed by an experienced resident and reviewed by an experienced chief physician. The annotated results were used for training the target detection network (Neural Network A). Neural Network A (Figure 3b) employed YOLOX-L architecture [20], a potent and high-speed object detection network. It was trained using the training parameters of YOLOX-L (https://github.com/Megvii-BaseDetection/YOLOX accessed on 20 January 2022) for 450 epochs (optimizer: SGD, momentum: 0.9, weight decay: 0.0005, maximum learning rate: 0.010, confidence threshold: 0.25), enabling it to detect the target ROI accurately. Neural Network A can process bitmap images in PNG or JPG format. Using the trained Neural Network A, all of the frontal radiographs in Dataset A and Dataset B were detected without any failures, and all results met the above-mentioned criteria for the ROI.

### 2.3. Image Processing Module

The EOS imaging system is an advanced X-ray imaging technology that not only enables the capture of high-quality images of the entire torso or even the entire human body in a single scan but also significantly reduces the radiation dose compared to traditional X-ray approaches. It allows for the simultaneous acquisition of frontal and lateral 2D images, which are captured orthogonally and spatially calibrated to each other [21]. Based on the characteristic of EOS imaging aligned in frontal and lateral views, the ROI in the lateral view can be obtained by aligning the corresponding frontal ROI in terms of height (Figure 3c). The image processing module cropped and stitched the ROI detected in the frontal view and the corresponding aligned ROI in the lateral view, based on the coordinate output by Neural Network A, to obtain a square image containing the biplanar ROIs (Figure 3d), which served as the input image for the subsequent neural network.

### 2.4. Neural Network B: Classification Network

Neural Network B is a three-classification network (Figure 3e) that we built using the EfficientNetV2-M framework [22]. It categorizes pre-menarche females, post-menarche females, and males. EfficientNetV2 introduces techniques like Fused-MBConv and is a well-designed, fast convolutional neural network for image recognition. It offers faster training speed, higher accuracy, and better parameter efficiency compared to previous models [22]. The ground truth labels were collected based on the actual conditions, while the input consisted of images with biplanar ROIs processed by the image processing module. To evaluate the neural network, we employed a five-fold cross-validation strategy, randomly dividing Dataset A into five mutually exclusive subsets (Table 1). For each iteration, one subset was used as the test set, while the remaining four subsets were merged and used as the training set to train the model. The training parameters were based on the ImageNet21K dataset (https://github.com/google/automl/tree/master/efficientnetv2 accessed on 20 January 2022), and we adopted the AdamW optimizer [23]. Each iteration involved training for approximately 120 epochs (maximum learning rate: 0.00035, betas: [0.9, 0.999], eps: 1e-6, width: 1.0, depth: 1.0, dropout: 0.3). Subsequently, the trained network was used to test the images in the corresponding test set, and the results were compared against the ground truth.

### 2.5. Integrated AI Program

The trained Neural Network A, Neural Network B (Iteration #1), and the intermediate image processing module were packaged and integrated into a standalone AI program, whereby the operation process of which is shown in Figure 3a–e. This AI program can be deployed in Windows or Linux systems and the final results can be generated with a one-click operation using the input of biplanar EOS images.

### 2.6. Clinical Validation

The integrated AI program was deployed in an environment consisting of Xeon Gold 6142 + Nvidia RTX A6000 + Windows 10. We utilized this AI program for successive detection of all EOS images in Dataset B and subsequently compared the detection results with the actual situation.

### 2.7. Statistics

Statistics in this study were calculated using corresponding packages and functions in the Python programming language. For the three-classification issue, the confusion matrix was used to analyze the classification result of our algorithm on different categories, accuracy was used to evaluate the overall performance of the algorithm, and macro recall, macro precision, and macro F1 were used to evaluate the overall performance of the algorithm on all categories. For the binary classification issue, accuracy, sensitivity, specificity, and the area under the receiver operating characteristic (ROC) curve (AUC) were used to evaluate the performance of the classifier, and Cohen’s kappa coefficient was used to analyze the agreement between the algorithm and the actual situation. A kappa value ≥0.75 represents strong agreement, 0.4–0.75 represents moderate agreement, and ≤0.4 represents poor agreement.

## 3. Results

**Figure 4:**
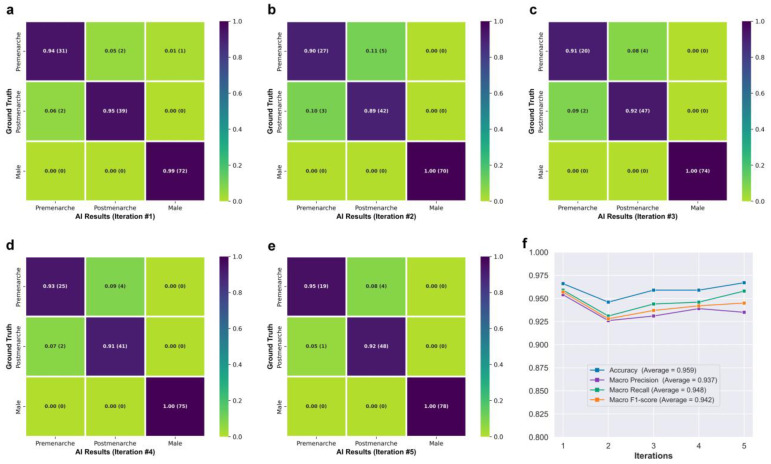
(**a**–**e**) Confusion matrices obtained from five iterations of testing. (**f**) Line graph showing the network’s performance.

Figure 3f shows the class activation maps for different categories of Neural Network B. For post-menarcheal females, the main basis for the network’s judgment is the diffuse image region around the pelvis and the thoracolumbar spine. For pre-menarcheal females, the network’s judgment is mainly based on the image region of the external genitalia and the lumbar vertebrae. For males, the network’s judgment is mainly based on the relatively limited biplanar image region near the external genitalia. Figure 4 shows the test results of Neural Network B using the five-fold cross-validation strategy. The confusion matrices obtained from the five iterations showed similar results, with the majority of misclassifications occurring between the pre-menarche and post-menarche groups. The accuracy of the five iterations was 0.966, 0.946, 0.959, 0.959, and 0.967, respectively. In addition, the macro F1-scores were observed to be 0.957, 0.928, 0.937, 0.942, and 0.945. Furthermore, the macro precision exhibited a range from 0.926 to 0.954, while the macro recall showcased a range spanning from 0.931 to 0.959.

**Figure 5:**
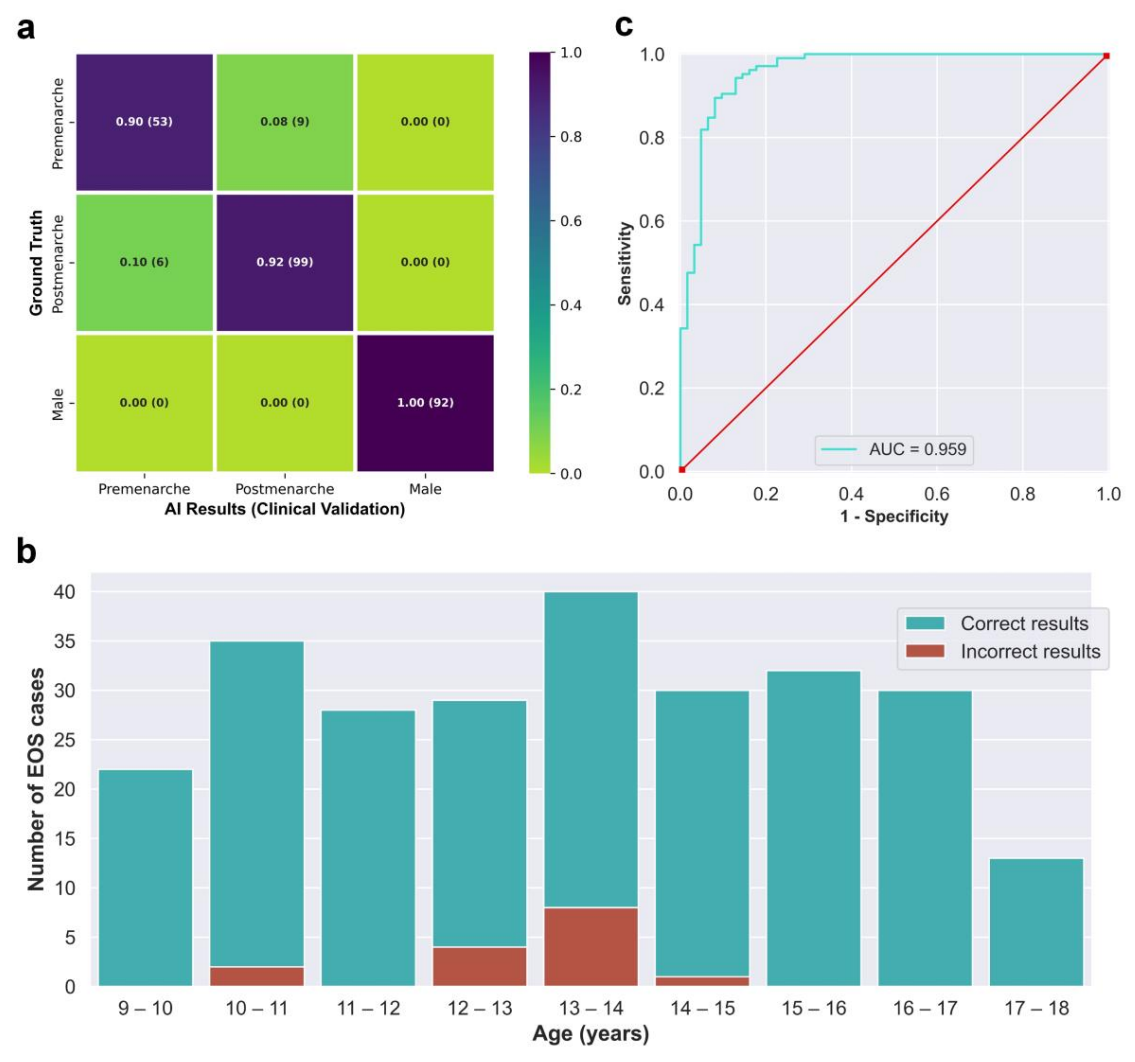
(**a**) Confusion matrix obtained from testing the integrated AI program on the clinical validation dataset. (**b**) The correct and incorrect results of the integrated AI program for different age groups. (**c**) Receiver operating characteristic curve of the integrated AI program for the identification of menarche status in females.

The integrated AI program was used to evaluate the 259 EOS cases from the clinical test set (Dataset B) in a continuous detection manner, which took a total of 119 s. The outcomes of the evaluation are visually displayed in the confusion matrix in Figure 5a. The matrix presents several key performance indicators, including accuracy of 0.942, macro precision of 0.933, macro recall of 0.938, and a macro F1-score of 0.935. The algorithm correctly identified males and females without any errors, while all detection errors occurred between the pre-menarche and post-menarche groups. The correct and incorrect identification results of different ages are depicted in Figure 5b, with most identification errors occurring between the ages of 12 and 14 years. When considering only females, the algorithm’s accuracy for identifying menarche status was 0.910, sensitivity was 0.943, specificity was 0.855, and the AUC calculated based on the ROC curve was 0.959. Moreover, the kappa value between the AI results and the actual situation was 0.806.

## 4. Discussion

This study proposes a deep-learning-based method for automatically identifying adolescent menarche status using biplanar X-ray images. It is capable of filling in the missing menarche information in specific clinical scenarios. To our knowledge, there have been no previous examples of using X-ray images to infer menarche status. In the field of medicine, algorithms designed based on deep learning methods have been attempted in many clinical tasks, such as the identification of lung diseases [24], the detection of diabetic retinopathy [25], the pathological diagnosis of kidneys [26], brain tumor segmentation [27], and the detection of colon polyps [28]. These medical tasks are mostly designed to simulate the judgment of doctors using artificial intelligence, but as there are no objective standards available for judging menarche status based on X-ray images, clinical doctors are still unable to estimate menarche status effectively. Therefore, the implementation of our task in a way surpasses the ability of clinicians in a sense.

The purpose of extracting ROI is to remove the unnecessary background, reduce redundant information, and, to some extent, eliminate the differences caused by the X-ray capturing process, with the goal of improving the efficiency and accuracy of subsequent detection [29]. In this study, we selected the trunk section from the first thoracic vertebra level to the lower trochanter level as the ROI on the frontal radiograph. Our ROI detection network adopted the YOLOX-L structure. Similar object detection networks have performed well in detecting some complex targets [30,31,32]. For the straightforward task of detecting and localizing large targets belonging to a single category in this study, merely a small amount of annotation and training enabled Neural Network A to achieve almost perfect automated detection and localization ability. All of the standard full-spine EOS images collected in this study were successfully processed by Neural Network A to obtain regions of interest (ROIs) that adhered to the aforementioned criteria without any instances of detection failure or positioning deviation observed.

After the fusion of the detected ROI and the corresponding lateral region, the target image contains biplanar radiological information on body parts that undergo significant changes during puberty, such as external genitalia, pelvis, chest, and spine. The regular changes in these parts [33] theoretically have the potential to serve as a basis for assessing the menarche status based on X-ray, but it is quite troublesome and difficult to manually analyze the radiological features of these parts, and to our knowledge, there have been no attempts in this regard. To address this challenge, we built a classification network, Neural Network B, based on the EfficientNetV2-M framework [22]. Convolutional neural networks that share similar structures with EfficientNetV2-M have demonstrated robust performance in various medical classification tasks [34,35,36].

Although both Dataset A and Dataset B were obtained through retrospective collection methods, they exhibit discernible dissimilarities in terms of age and gender distributions, as depicted in Figure 2. Dataset A, which serves as the primary data source for network training and internal testing purposes, required a balanced distribution. Consequently, we imposed quantity restrictions on each subgroup within Dataset A. On the other hand, Dataset B consists of consecutive cases in our clinical practice and offers a more authentic representation of clinical scenarios.

The classification of menarche status is the primary goal of this study. To ensure broader applicability and prevent errors when encountering male cases, thereby accommodating the entire target population, we included a “male” classification in the design of Neural Network B, making it applicable to all age-appropriate populations. The class activation maps in Figure 3f demonstrate that Neural Network B can effectively capture the differences in external genitalia between males and females, as well as the changes in external genitalia, pelvis, and spine in females during growth and development. In the case of post-menarcheal females, the network primarily relies on diffuse image regions around the pelvis and thoracolumbar spine for classification. In the case of pre-menarcheal females, the network primarily relies on image regions related to the external genitalia and the lumbar vertebrae. In the process of five-fold cross-validation, the evaluation of model performance yielded a range of macro F1-scores from 0.928 to 0.957, accompanied by accuracy scores spanning from 0.946 to 0.967. The approximate results obtained from the five iterations serve as evidence supporting the stability and robustness of this methodology. Additionally, during the subsequent clinical validation, specifically for the three-class classification task, the algorithm achieved a macro F1-score of 0.935. Despite differences in category and age composition between Dataset A and Dataset B, the algorithm achieved similar results in clinical validation as those obtained from the five-fold cross-validation, as shown by the confusion matrix and related results. Our algorithm rarely made errors in gender identification, and even in clinical validation, no errors were made. As shown in Figure 5b, the main errors of the algorithm occurred in the age range of 12 to 14, which includes the average age of menarche for Chinese adolescent females [37]. Reasonably, the menarche status in females who are close to their first menstruation is more difficult to determine from X-ray images compared to females of other ages. This phenomenon indirectly reflects the similarities between this algorithm and human cognition. An improvement in future algorithm accuracy is essential in order to address this issue. Additionally, in the assessment of menarche status of females, the algorithm achieved an AUC of 0.959 and a kappa value of 0.806, indicating strong agreement between the algorithm and the real-world scenario.

In testing, the integrated AI program completed the identification of one EOS case in an average time of only 0.46 s, and with iterative upgrades in computer hardware levels in the future, the required time will be further compressed. The results obtained by this program may assist doctors in making decisions in certain specific clinical situations, and the cost of this process in terms of manpower and time is almost negligible.

There are several limitations to this study that should be acknowledged. First, the amount of data used in this study is relatively limited compared to some large computer vision task datasets [38,39,40]. Therefore, we employed a five-fold cross-validation strategy during the training and testing of the classification neural network (Dataset A) to maximize data utilization and ensure a thorough evaluation of its performance. Additionally, we further validated the classification performance of the integrated AI program by incorporating an additional clinical validation set (Dataset B). Second, data from one single center and one single ethnicity may also limit the generalizability of our method. Third, it is worth noting that this study did not account for the occurrence of extreme variations in menarche associated with conditions like serious illnesses or developmental abnormalities. Despite these, this study still demonstrates the advantages of deep learning strategies in processing medical image data to some extent, and provides a convenient method for assessing the status of menarche. Certainly, our study represents an initial step, and more accurate algorithms will require extensive data support from multiple centers and diverse populations in the future.

## 5. Conclusions

This study proposed an automated deep-learning-based method that can efficiently and conveniently identify the menarche status of adolescent patients based on EOS X-ray images. It is expected to become a routine clinical tool that can assist clinicians in diagnosis and treatment under specific clinical conditions. In this study, the algorithm model achieved robust generalization ability with merely a limited amount of training data. Before actual clinical application in the future, further research is required to improve its ability to handle special cases and further enhance its accuracy.

## Figures and Tables

**Table 1:** Details and basic characteristics of Dataset A.

			Female								Male				Total			
			Pre-menarche				Post-menarche											
			P_25_	Median	P_75_	Count	P_25_	Median	P_75_	Count	P_25_	Median	P_75_	Count	P_25_	Median	P_75_	Count
Subsets	1	Age (years)	9.9	10.7	11.8	34	13.8	14.6	16.5	41	11.2	13.9	15.8	72	11.1	13.6	15.7	147
	2	Age (years)	9.8	10.8	11.6	32	13.3	14.8	16.2	45	11.3	13.6	15.6	70	11.1	13.4	15.4	147
	3	Age (years)	9.7	10.9	11.8	24	13.8	15.1	16.4	49	11.1	12.7	15.7	74	11.2	13.4	15.5	147
	4	Age (years)	10.1	10.9	11.6	29	14	15.5	16.4	43	11.3	13	15.3	75	11.2	13.2	15.6	147
	5	Age (years)	9.5	10.7	11.8	23	13.8	15.6	16.7	49	10.8	13.3	16.3	78	11.3	13.8	16.3	150
Total		Age (years)	9.8	10.8	11.8	142	13.8	15.1	16.4	227	11.1	13.5	15.7	369	11.2	13.5	15.7	738

Abbreviation: P_25_, 25th percentile; P_75_, 75th percentile.

## Data Availability

Currently, the datasets generated and analyzed during the current study cannot be made publicly accessible due to privacy protection.
